# Direct inhibition of dioxygenases TET1 by the rheumatoid arthritis drug auranofin selectively induces cancer cell death in T-ALL

**DOI:** 10.1186/s13045-023-01513-6

**Published:** 2023-11-22

**Authors:** Long Chen, Anqi Ren, Yuan Zhao, Hangyu Chen, Qifang Wu, Mengzhu Zheng, Zijian Zhang, Tongcun Zhang, Wu Zhong, Jian Lin, Haichuan Zhu

**Affiliations:** 1 Department of Pharmacy, Peking University Third Hospital Cancer Center, Peking University Third Hospital, Peking University, Beijing, 100191 China; 2 Institute of Biology and Medicine, College of Life and Health Sciences, Wuhan University of Science and Technology, Wuhan, 430081 China; 3 Key Laboratory of Tropical Biological Resources of Ministry of Education, School of Pharmaceutical Sciences, Song Li’s Academician Workstation of Hainan University, Hainan University, Sanya, 572000 China; 4 National Engineering Research Center for the Emergency Drug, Beijing Institute of Pharmacology and Toxicology, Beijing, 100850 China; 5 Synthetic and Functional Biomolecules Center, Peking University, Beijing, 100871 China

**Keywords:** DNA methylation, TET1, Auranofin, 5hmC, c-Myc

## Abstract

T-cell acute lymphoblastic leukemia (T-ALL) is a type of hematologic tumor with malignant proliferation of hematopoietic progenitor cells. However, traditional clinical treatment of T-ALL included chemotherapy and stem cell transplantation always lead to recurrence and poor prognosis, thus new therapeutic targets and drugs are urgently needed for T-ALL treatment. In this study, we showed that TET1 (ten-eleven translocation 1), a key participant of DNA epigenetic control, which catalyzes the conversion of 5-methylcytosine (5mC) to 5-hydroxymethylcytosine (5hmC) to modulate gene expression, was highly upregulated in human T-ALL and negatively correlated with the prognosis of patients. Knockdown of TET1 suppressed T-ALL growth and progression, suggesting that TET1 inhibition maybe an effective way to fight T-ALL via DNA epigenetic modulation. Combining structure-guided virtual screening and cell-based high-throughput screening of FDA-approved drug library, we discovered that auranofin, a gold-containing compound, is a potent TET1 inhibitor. Auranofin inhibited the catalytic activity of TET1 through competitive binding to its substrates binding pocket and thus downregulated the genomic level of 5hmC marks and particularly epigenetically reprogramed the expression of oncogene c-Myc in T-ALL in TET1-dependent manner and resulted in suppression of T-ALL in vitro and in vivo. These results revealed that TET1 is a potential therapeutic target in human T-ALL and elucidated the mechanism that TET1 inhibitor auranofin suppressed T-ALL through the TET1/5hmC/c-Myc signaling pathway. Our work thus not only provided mechanism insights for T-ALL treatment, but also discovered potential small molecule therapeutics for T-ALL.

**Supplementary Information:**

The online version contains supplementary material available at 10.1186/s13045-023-01513-6.

To the editor,

TETs is diversely expressed in hematological malignancies and serves as potential therapeutic targets [1–5]. However, the roles of TET1 in T-ALL have not been fully unveiled, and potent TET1 inhibitors are needed to promote TET1 as a druggable target [6–8].

We analyzed gene expression from previous T-ALL cohorts and found TET1, but not TET2/3, is highly expressed in T-ALL and drug-resistant patients (Fig. 1a, b, Additional file 2: Fig. S1a–c). Validation in T-ALL cell lines confirmed TET1 upregulation in T-ALL cells compared to normal T cells and further upregulation in dexamethasone-resistant cells (Fig. 1c). TET1 expression negatively correlates (even though not significant) with poor prognosis (Fig. S1d). Silencing of TET1 impaired the proliferation of T-ALL cells (Fig. 1d, Additional file 2: Fig. S1e–i), indicating crucial roles of TET1 in T-ALL. However, treatment of T-ALL cells with two reported TET1 inhibitors only showed minimum proliferation inhibition (Additional file 2: Fig. S2), due to low potency of the inhibitors 6, suggesting more potent TET1 inhibitors are needed.

**Fig. 1:**
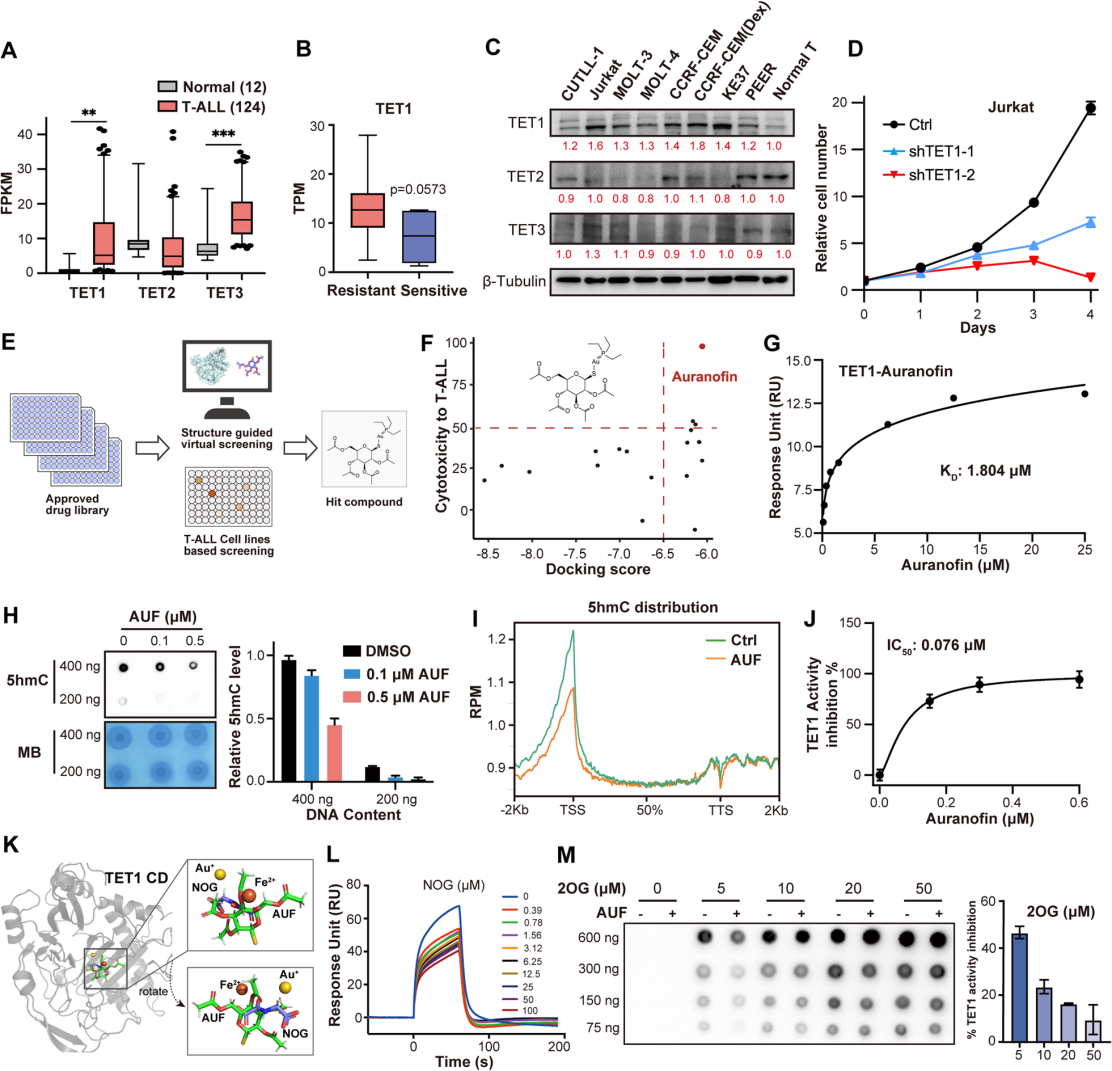
**a** Fragments Per Kilobase Million (FPKM) of TET1, TET2, TET3 in a cohort with 124T-ALL patients and 12 normal T cell samples (CNCB, HRA000122). Data are mean ± SD (Two-tailed unpaired Student’s t test, ** *p* < 0.01, *** *p* < 0.001).** b** Transcripts per million (TPM) of TET1 in glucocorticoid-resistant and glucocorticoid-sensitive T-ALL samples (GSE5820). **c** Protein expression levels of TET1, TET2, TET3 in 8 T-ALL cell lines and normal T cells. **d** Effect of TET1 knock down on cell growth of Jurkat cell line. **e** Schematic of structure guided virtual screening and cell line-based drug screening with 2059 FDA or EMA approved drugs. Jurkat and CCRF-CEM cell lines were treated with each drug (5 μM) for 48 h, and cell viability was detected using CCK-8 assay. **f** Joint analysis of virtual screening and cell line-based screening identified auranofin as a potential hit drug. **g** Affinity between TET1 protein and auranofin assayed by surface plasmon resonance (SPR). Equilibrium binding analysis indicates a K_D_ of 1.804 μM. **h** Dot blot analysis of global 5hmC in Jurkat cell showing that auranofin dose-dependently decreased cellular 5hmC level. Left: dot blot image; right: quantification of dot blot results. **i** Quantification of genome-wide 5hmC distribution in T-ALL Jurkat cells treated with or without 0.1 μM auranofin for 24 h. Auranofin treatment decreased global 5hmC levels. *n* = 5 replicates. **j** Dose-dependent inhibition of TET1 catalytic activity by auranofin with an in vitro fluorometric quantification assay. IC_50_ of 0.076 μM was determined, indicating potent inhibition of TET1 by auranofin. **k** Molecular docking showing that auranofin are in close conformation with 2-OG and Fe (II) in the binding pocket of TET1-CD. **l** SPR-based assay showing that the binding of auranofin to TET1 was competed by increasing concentration of TET1 substrate analogue NOG. **m** Dot blot analysis of 5hmC revealed that auranofin induced TET1 catalytic activity inhibition was attenuated by increasing concentration of 2-OG. Left: dot blot image; right: quantification of dot blot results

To identify potent TET1 inhibitors, we performed virtual- and cell-based screening (Fig. 1e). Auranofin was identified from both screening (Fig. 1f, Additional file 1: Table S1–S2). SPR revealed that auranofin binds to TET1 (Fig. 1g), with reduced affinity to TET2/3 (Additional file 2: Fig. S3).

Methylation-related pathways were enriched after auranofin treatment in T-ALL (Additional file 2: Fig. S4a, b, Additional file 1: Table S3), which induced dose-dependent decrease in cellular 5hmC and increase in 5mC, as indicated by dot blot and genome-wide 5hmC/5mC sequencing (Fig. 1h-i, Additional file 2: Figs. S4c, S5). LC–MS/MS confirmed inhibition of TET1 catalyzed 5mC to 5hmC conversion by auranofin (Additional file 2: Fig. S6). In vitro assay determined a IC_50_ of 76 nM (Fig. 1j).

We generated structure of TET1-CD-auranofin by molecular docking to explore the inhibition mechanism (Fig. 1k). Structure showed that auranofin was in very close conformation with NOG (analog of TET1 substrate 2-OG) and Fe (II), suggesting a potential inhibition mechanism of auranofin through competitively preventing substrates binding to TET1. Results indicated that both NOG and Fe (II) compete with auranofin for binding to TET1-CD and increasing concentration of 2-OG or Fe (II) attenuated auranofin inhibition on TET1 (Fig. 1l-m and Additional file 2: Fig. S7). Given that, 2-OG and Fe (II) are conserved in many demethylases, selectivity of auranofin to TET1 was verified by comparing to TET2 and KDM6B. Auranofin showed reduced affinity and very low inhibition at 1 μM to both proteins (Additional file 2: Fig. S8), indicating at least 13-fold selectivity for TET1 over TET2 and KDM6B. Structure and electrophoretic mobility shift assay also showed that auranofin did not affect DNA substrate binding to TET1 (Additional file 2: Fig. S9). Collectively, auranofin inhibits TET1 by competing with substrates for binding to TET1.

Auranofin was highly cytotoxic to T-ALL cells, but not to normal T cells (Fig. 2a). Overexpressing either full length or the catalytic domain of TET1, but not the catalytic dead mutants or TET2/TET3-CD, in T-ALL attenuated the cytotoxicity of auranofin (Fig. 2b, c and Additional file 2: Figs. S10–S12), revealing that the auranofin-mediated cytotoxicity depends on the catalytic activity of TET1. In vivo T-ALL xenograft model showed that treatment with auranofin significantly inhibited progression, as well as bone marrow invasion, of T-ALL and prolonged mice survival (Fig. 2d–i), indicating therapeutic potential of auranofin for T-ALL.

**Fig. 2:**
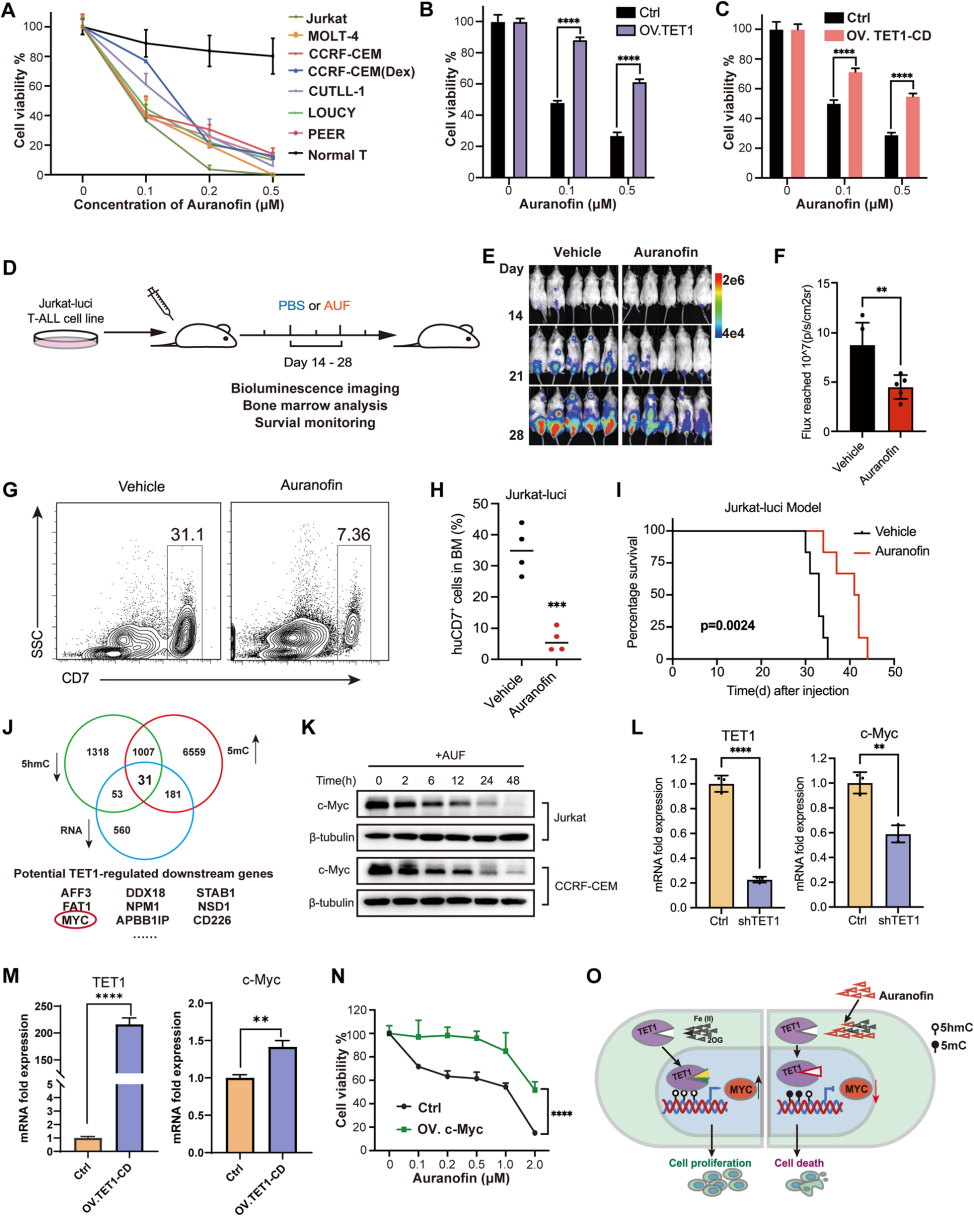
**a** Relative cell growth of T-ALL cell lines and normal T cell after treatment with various concentration of auranofin. Cell numbers were counted at 48 h post treatment with auranofin. **b** Auranofin-induced T-ALL cell death was attenuated by full-length TET1 overexpression. Data are mean ± SD (Two-tailed unpaired Student’s t test, **** *p* < 0.0001). **c** Auranofin-induced T-ALL cell death was attenuated by TET1-CD overexpression. Data are mean ± SD (Two-tailed unpaired Student’s t test, **** *p* < 0.0001). **d** Schematic of cell-derived xenograft (CDX) validating the in vivo anti-T-ALL activity. A total of 5 × 10^6^ luciferase-expressing Jurkat cells were injected through the tail vein to NCG mice. Auranofin (20 mg/kg/2 days) was intraperitoneally administered from day 14 to day 28 with PBS as Vehicle. **e** Time-lapse bioluminescence imaging of the xenograft mice. **f** Quantification of the total photon flux of the xenograft mice at day 28. Data are mean ± SD (Two-tailed unpaired Student’s t test, ** *p* < 0.01). **g** Representative flow cytometry analysis of bone marrow resident Jurkat cancer cells in Vehicle and auranofin treated mice. Jurkat cancer cells were stained with anti-human CD7 antibody. **h** Quantification of the bone marrow resident Jurkat cancer cells shown in g. Two-tailed unpaired Student’s t test, *** *p* < 0.001. **i** Kaplan–Meier survival curves the xenograft mice treated with Vehicle or auranofin. **j** Venn diagram depicting vital genes which are intersected among 5hmC and RNA downregulated and 5mC upregulated genes. 31 intersected genes including c-Myc were identified. **k** Western blot analysis showing the time-dependent down-regulation of c-Myc expression in Jurkat and CCRF-CEM cells. **l** qRT-PCR analysis showing that c-Myc mRNA decreased after knockdown of TET1. **m** qRT-PCR analysis showing that c-Myc mRNA increased after overexpression of TET1. **n** Auranofin-induced T-ALL Jurkat cell death was attenuated by c-Myc overexpression at various auranofin concentrations. Data are mean ± SD (Two-tailed unpaired Student’s *t* test, **** *p* < 0.0001). **o** Schematic diagram showing the proposed mechanism of action of auranofin in T-ALL. Auranofin inhibits TET1 enzymatic activity through competitive occupation of the binding pocket to its cofactor substrates 2-oxoglutarate (2-OG) and Fe (II). Inhibition of TET1 subsequently down-regulated the transcription and translation of c-Myc, at least partially through c-Myc DNA epigenetic remodeling, leading to T-ALL cells death

Auranofin has been reported to exert anti-tumor activity via Thioredoxin reductases and ROS [9, 10]. While, mechanism in T-ALL is different as neutralizing ROS or genetic manipulation of Thioredoxin reductases did not affect auranofin cytotoxicity to T-ALL (Additional file 2: Fig. S13).

Auranofin treatment altered genome-wide distribution of 5hmC/5mC in the promoter region (Fig. 1i, Additional file 2: Fig. S5, Additional file 1: Tables S4, S5), enlightened potential mechanism of action of auranofin via epigenetic control of transcription and translation of certain genes. Conjointly analysis of 5hmC-Seal, WGBS, and RNA-Seq data, identified 31 genes with 5hmC/RNA down-regulation and 5mC up-regulation, among which c-Myc, a central oncogene in T-ALL [11, 12], was discovered (Fig. 2j, Additional file 2: Fig. S14, Additional file 1: Table S6). Auranofin treatment down-regulated both c-Myc transcription and translation (Fig. 2k, Additional file 2: Fig. S15a, b). TET1 correlates with c-Myc expression (Additional file 2: Fig. S15c, d), as validated by genetic manipulation of TET1 (Fig. 2l–m). Overexpression of c-Myc in T-ALL attenuated auranofin-induced cytotoxicity (Fig. 2n, Additional file 2: Fig. S15e), suggesting c-Myc as downstream effector.

Collectively, we confirmed TET1 is a promising therapeutic target for T-ALL and discovered potent TET1 inhibitor, auranofin, with anti-T-ALL activity in vitro and in vivo. Mechanistically, auranofin-induced TET1 inhibition epigenetically alters transcription and translation of c-Myc to induce T-ALL cell death (Fig. 2o).

### Supplementary Information

**Additional file 1**. Supplementary tables.

**Additional file 2**. Supplementary figures.

## Data Availability

The RNA-seq, 5hmc-seq and WGBS data in this study have been deposited in the Genome Sequence Archive (GSA) for human under accession number HRA004879, which is publicly available. The transcriptomic of T-ALL patients cohort was retrieved from Genome Sequence Archive (GSA) for human under accession number HRA00122 which our previous published. The transcriptomic and clinical data of T-ALL patients cohort was retrieved from TARGET, phs000464. GEO accession codes of the published data used in this study are as follows: The data of glucocorticoid resistance in T-ALL patients, GEO: GSE5820; The data of T-ALL samples compared to normal BM samples and correlation analysis of TET1 and c-Myc expression in T-ALL patients, GEO: GSE26713, GSE146901. This paper does not report original code. Any additional information required to reanalyze the data reported in this paper is available from the lead contact upon request.

## Abbreviations

T-ALL: T-cell acute lymphoblastic leukemia
TETs: TET family proteins (TET1, TET2, TET3)
TET1: Ten-eleven translocation 1
5mC: 5-Methylcytosine
5hmC: 5-Hydroxymethylcytosine
SPR: Surface Plasmon Resonance
NOG: N-Oxalylglycine
2-OG: 2-Oxoglutarate
TET1-CD: Catalytic domain of TET1
TET1-C2: Catalytic domain of TET2
TET3-CD: Catalytic domain of TET3
ROS: Reactive oxygen species
WGBS: Whole-genome bisulfite sequencing
