# Supplementary material for: Direct inhibition of dioxygenases TET1 by the rheumatoid arthritis drug auranofin selectively induces cancer cell death in T-ALL
Source: J Hematol Oncol. 2023 Nov 22;16:113. doi: 10.1186/s13045-023-01513-6 (PMC10666452; doi:10.1186/s13045-023-01513-6)
Supplement: Supplementary file 2 — Additional file 2. Supplementary figures. [file 13045_2023_1513_MOESM2_ESM.docx]

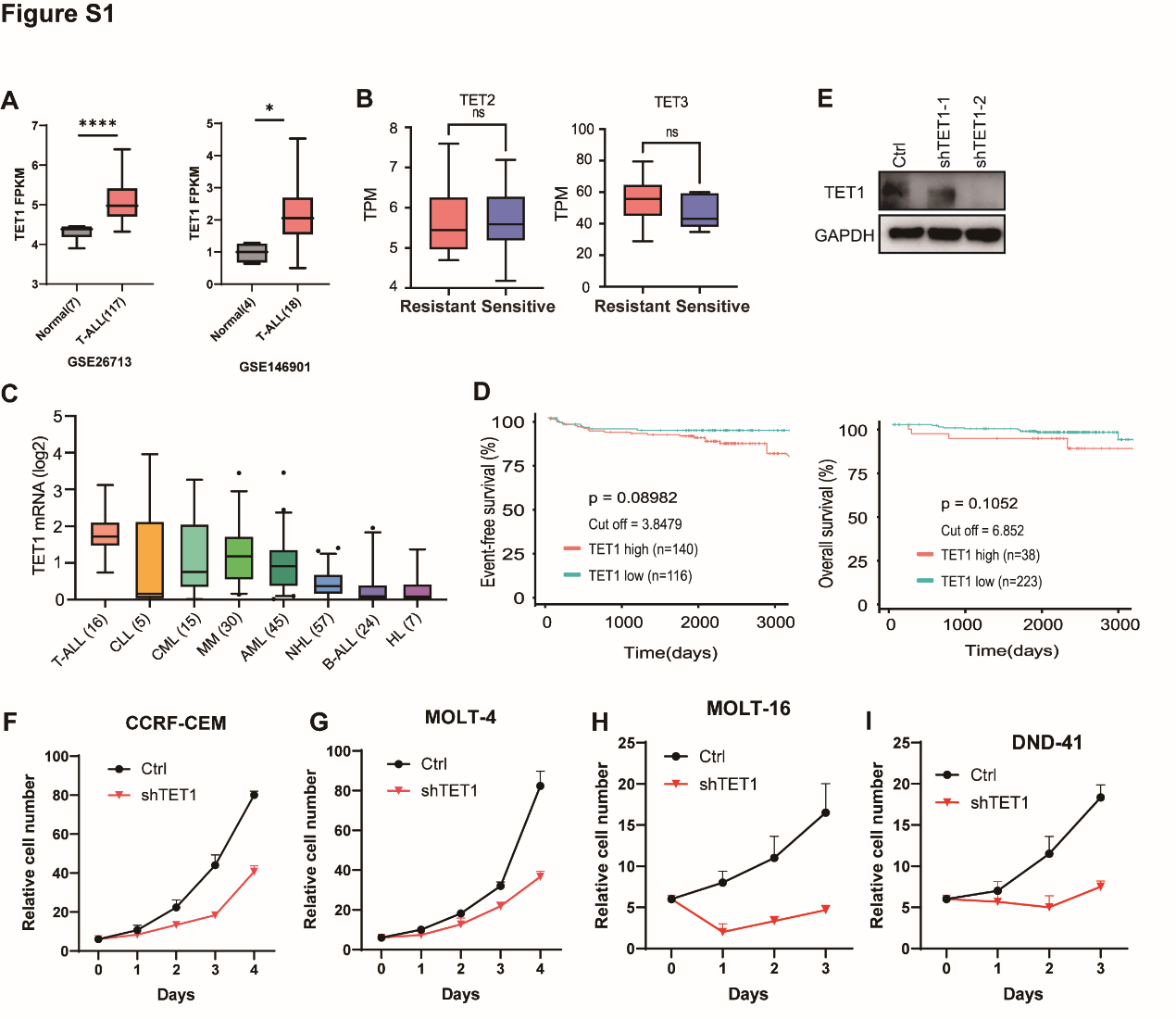


**Figure S1. a.** Fragments Per Kilobase Million (FPKM) of TET1 from datasets GSE26713 and GSE146901. **b.** Transcripts per million (TPM) of TET2, and TET3 in glucocorticoid-resistant and glucocorticoid-sensitive T-ALL samples from dataset GSE5820. **c.** mRNA expression levels of TET1 in different hematopoietic malignancies, data from the Cancer Cell Line Encyclopedia (CCLE, https://sites.broadinstitute.org/ccle/). **d.** Event-free survival and overall survival of T-ALL patients with higher or lower expression of TET1, data from TARGET, phs000464. **e.** Western blot analysis of TET1 expression in normal and TET1 knockdown Jurkat cells shown in main Figure 1D. **f.** Effect of TET1 knockdown on cell growth of CCRF-CEM. **g.** Effect of TET1 knockdown on cell growth of MOLT-4 cells. **h.** Effect of TET1 knockdown on cell growth of MOLT-16 cells. **i.** Effect of TET1 knockdown on cell growth of DND-41 cells.


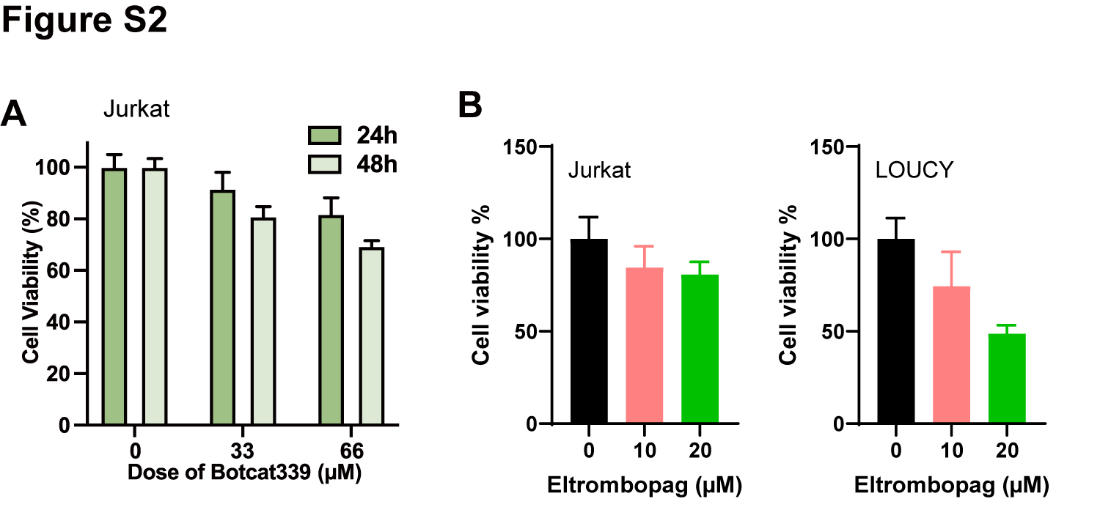


**Figure S2. a.** Jurkat cell viability treated with different concentrations of a previously reported TET inhibitor Bobcat339 for 24 and 48 hours. **b.** Cell viability of Jurkat (left) and LOUCY (right) T-ALL cells treated with a reported TET inhibitor Eltrombopag for 24 hours.


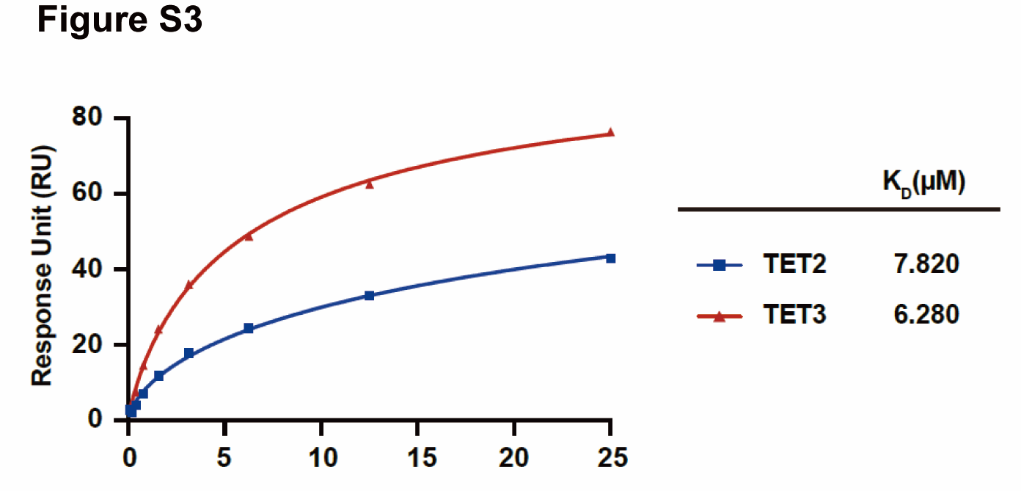


**Figure S3.** Affinity between TET2 or TET3 protein and auranofin assayed by surface plasmon resonance (SPR). Equilibrium binding analysis indicates a K_D_ of 7.820 μM and 6.280 μM for TET2-auranofin and TET3-auranofin, respectively.


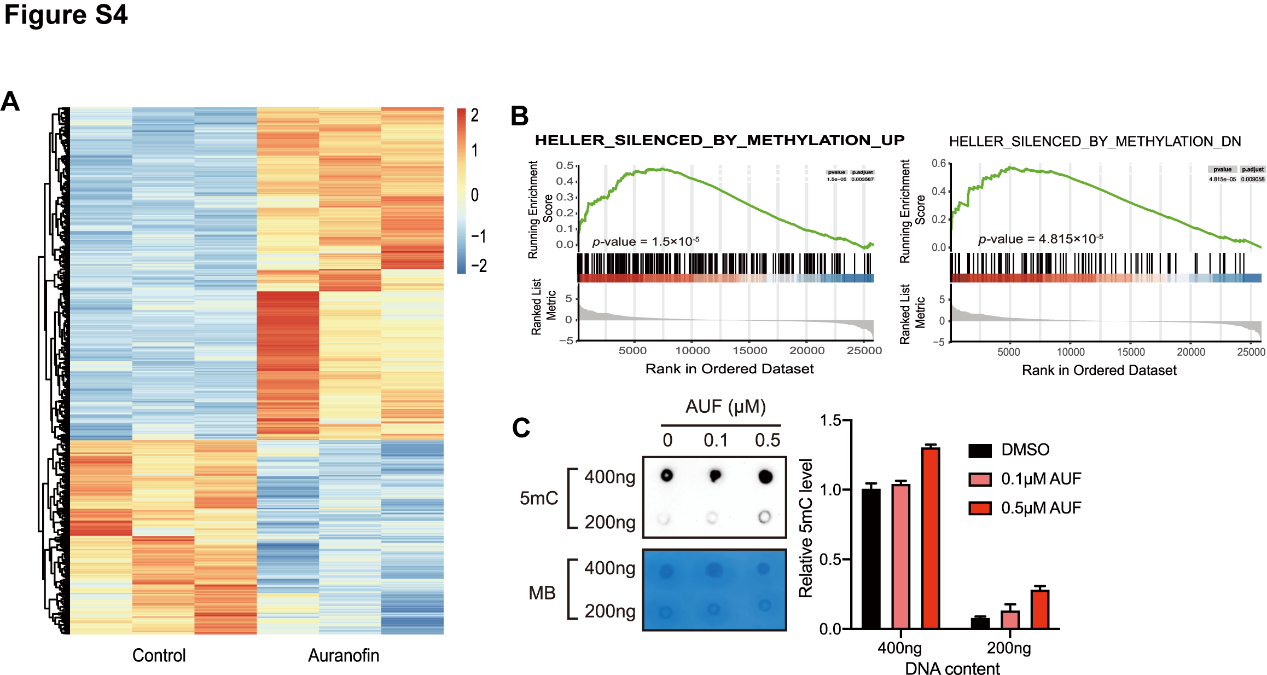


**Figure S4. a.** Heatmap of differentially expressed genes (DEGs) from RNA-Seq. Jurkat cell were treated with auranofin (0.1 μM) for 24 h and DEGs were filtered with p < 0.05 and |log2FoldChange| > 0.5. The colors indicate the Z score of the expression of different genes (Table S3). **b.** Gene set enrichment analysis (GSEA) of genes associated with methylation after auranofin treatment. **c.** Dot blot analysis of 5mC levels in Jurkat cells after treatment with different concentrations of auranofin (0.1 μM, 0.5 μM).


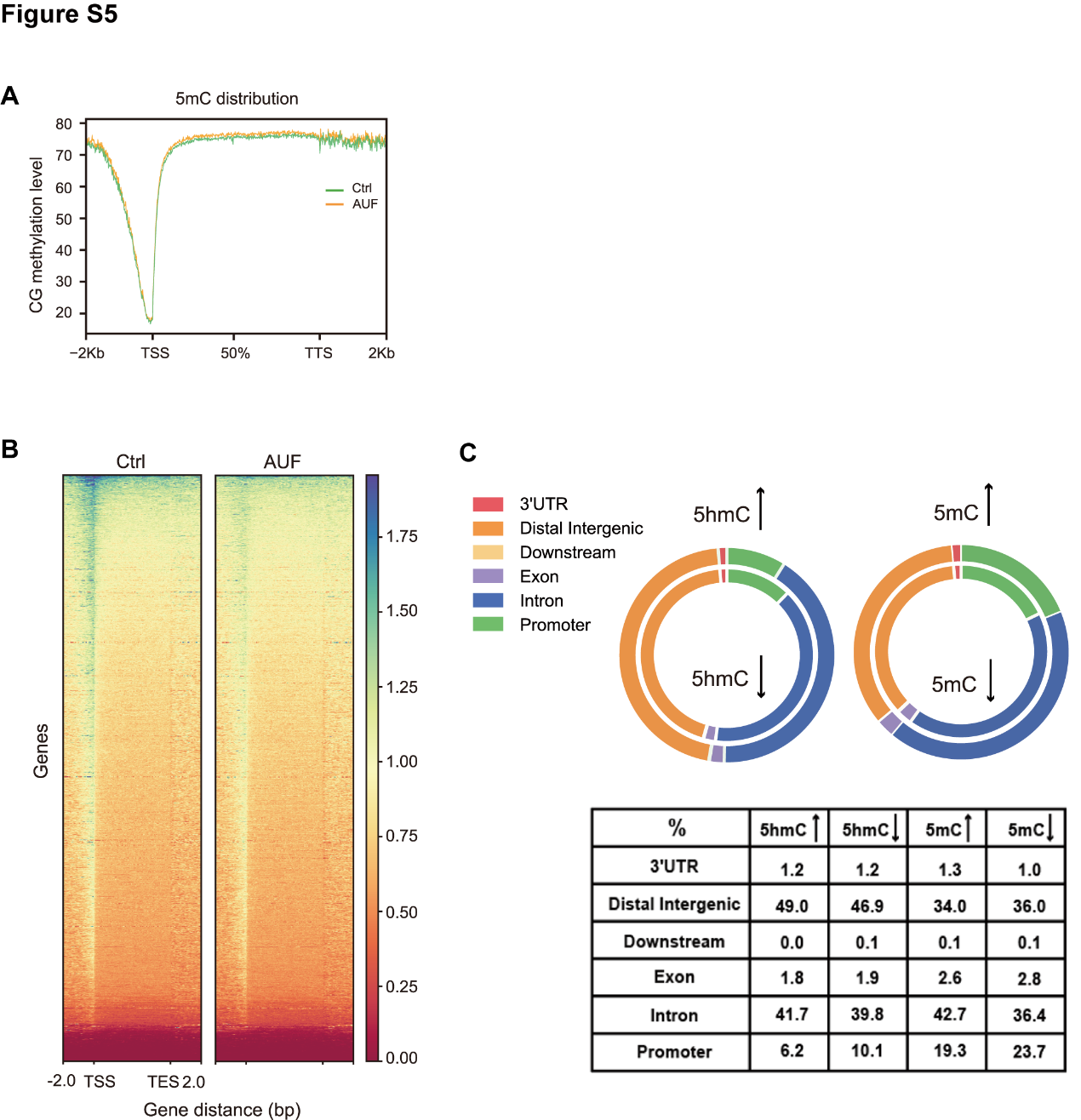


**Figure S5. a.** Quantification of global CG methylation levels in T-ALL cells treated with or without 0.1 μM auranofin for 24 h. n=5 replicates. **b.** Heatmap representation of 5hmC across TSSs and TTSs (±2 kb) in T-ALL cell lines after 24 h of auranofin (0.1 μM) treatment. n=5 replicates. Blue indicates enrichment, and yellow indicates no signal. **c.** Distribution of 5hmC and 5mC signals in the promotor, exon, intron, downstream, 3’UTR, and distal intergenic regions.


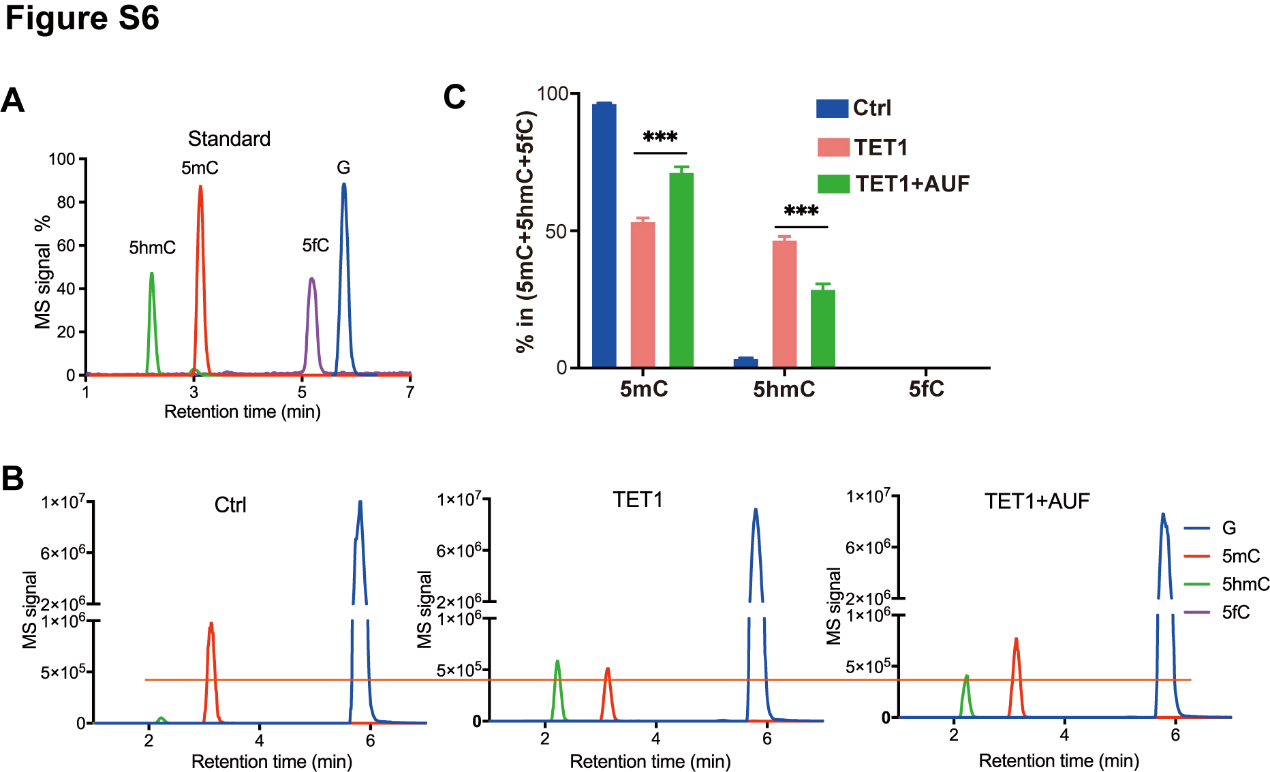


**Figure S6. a.** LC-MS/MS traces of different nucleoside standards. **b.** Quantification of 5hmC, 5mC, 5fC and G nucleosides from a synthetic single-stranded DNA, containing a single 5mC modification, catalyzed by TET1 with or without Auranofin inhibition. **c.** Quantification of 5mC, 5hmC and 5fC showing in b. Data are mean ± SD (Two-tailed unpaired Student’s t test, *** *p* < 0.001).


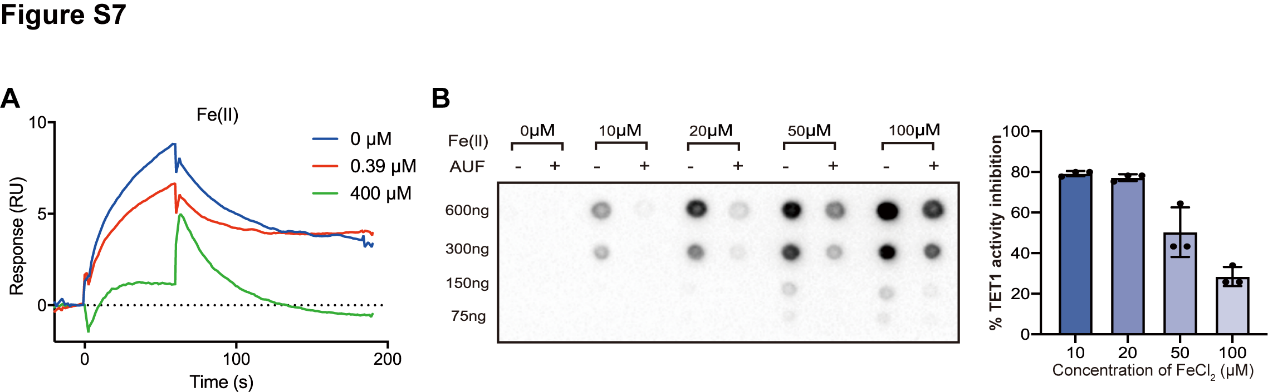


**Figure S7.** **a.** SPR-based assay showing that the binding of Auranofin to TET1 was competed by increasing concentration of TET1 cofactor Fe (II). **b.** Dot blot analysis of 5hmC revealed that auranofin induced TET1 catalytic activity inhibition was attenuated by increasing concentration of Fe (II). Left: dot blot image; right: quantification of dot blot results.


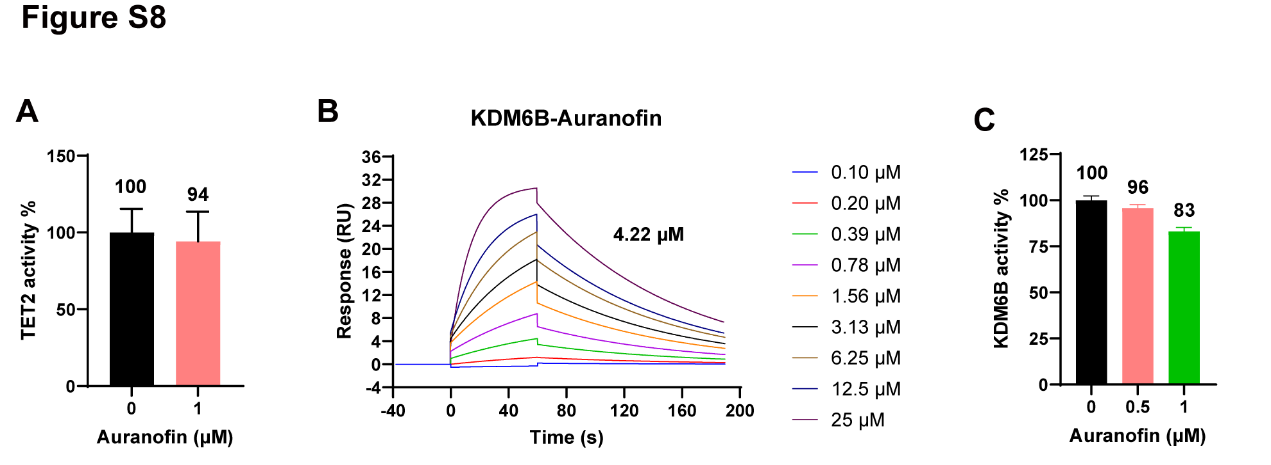


**Figure S8. a.** *In vitro* TET2 activity with or without the inhibition of 1 μM auranofin. Only 6% of TET2 activity was inhibited by 1 μM auranofin. **b.** Association of dissociation curves of auranofin binding to another 2-oxoglutarate (2OG) and Fe (II) dependent enzyme KDM6B. **c.** In vitro KDM6B activity with or without the inhibition of 0.5 or 1 μM auranofin. Only 17% of KDM6B activity was inhibited by 1 μM auranofin.


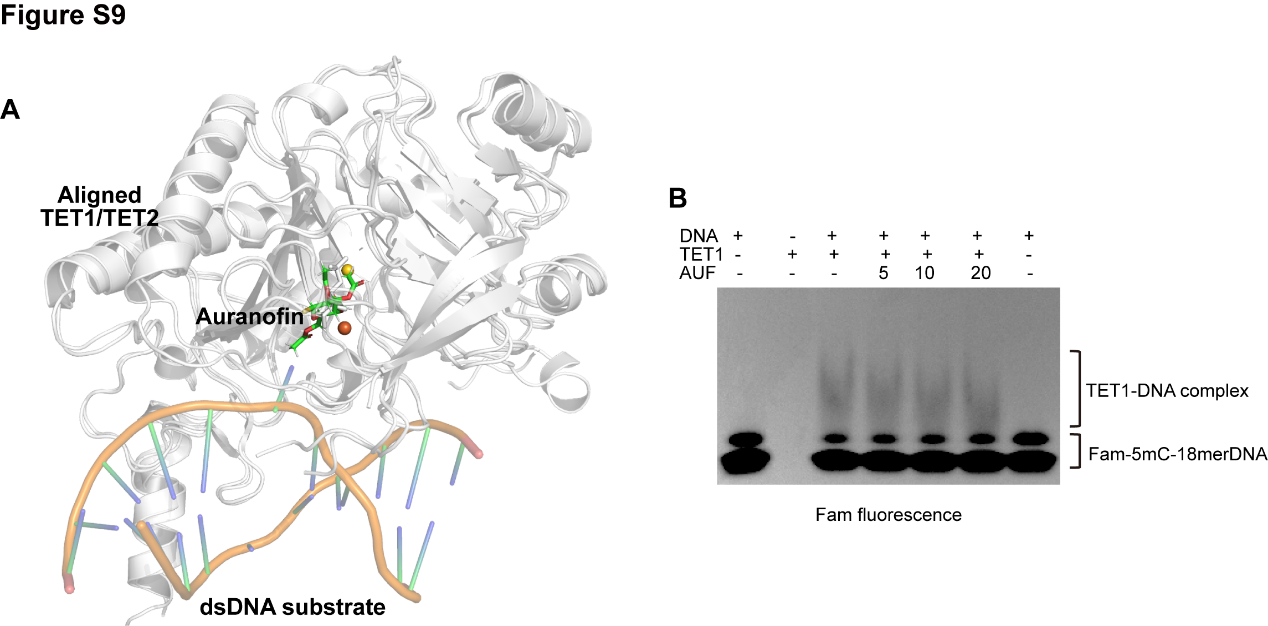


**Figure S9.** **a.** Molecular docking and alignment with TET1/2-DNA complex structure (PDB: 4NM6) showing the conformation of auranofin and DNA substrate. **b.** Electrophoretic mobility shift assay showing that the binding of a synthetic DNA substrate to TET1 was not altered by auranofin.


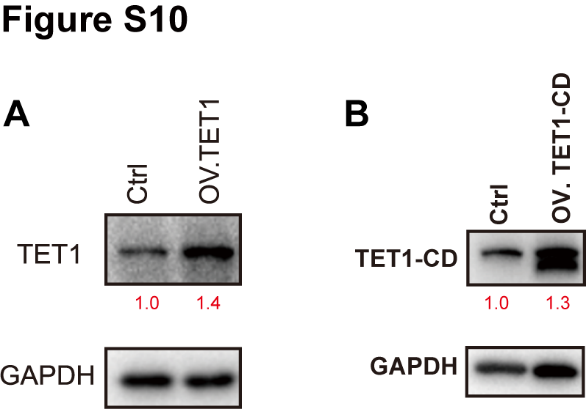


**Figure S10.** **a.** Western blot analysis showing the overexpression of full-length TET1. **b.** Western blot analysis showing the overexpression of the catalytic domain of TET1.


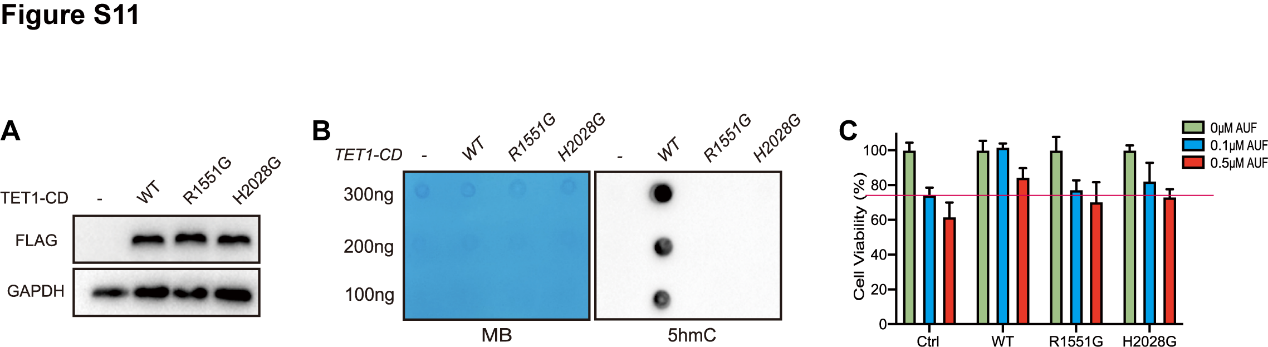


**Figure S11. a.** Western blot analysis of the overexpression of WT or mutant TET1-CD in Jurkat cells. **b.** Dot blot analysis of global 5hmC in Jurkat cells overexpressing WT or mutant TET1-CD. **c.** Viability of Jurkat cells overexpressing WT or mutant TET1-CD treated with different concentration of auranofin for 24 h. Overexpression of catalytic dead TET1-CD mutants did not attenuate auranofin-induced cytotoxicity.


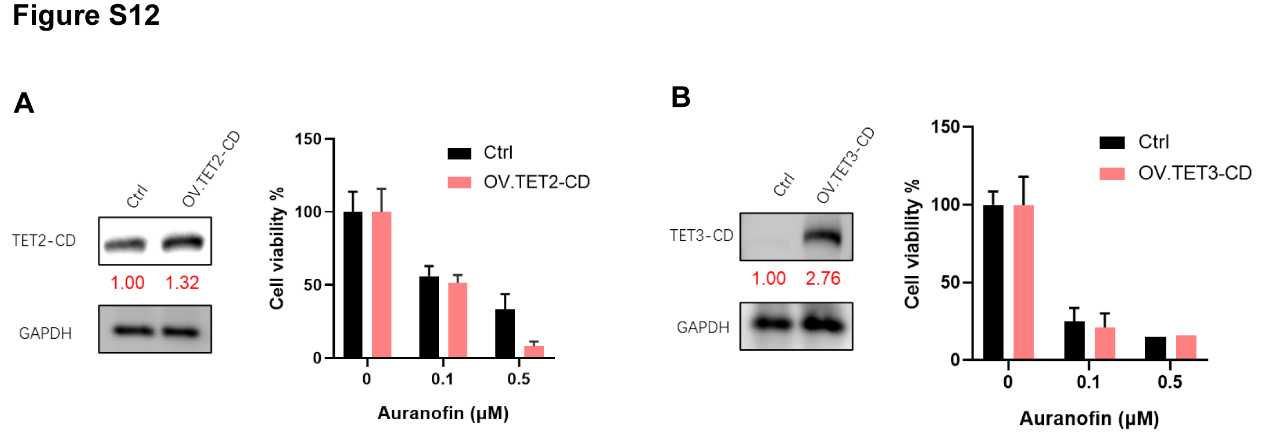


**Figure S12. a.** Cell viability of Jurkat cells with or without TET2-CD overexpression. Left: Western blot. Right: Cell viability. auranofin induced cell death was not rescued by TET2-CD overexpression. **b.** Cell viability of Jurkat cells with or without TET3-CD overexpression. Left: Western blot. Right: Cell viability. auranofin induced cell death was not rescued by TET3-CD overexpression.


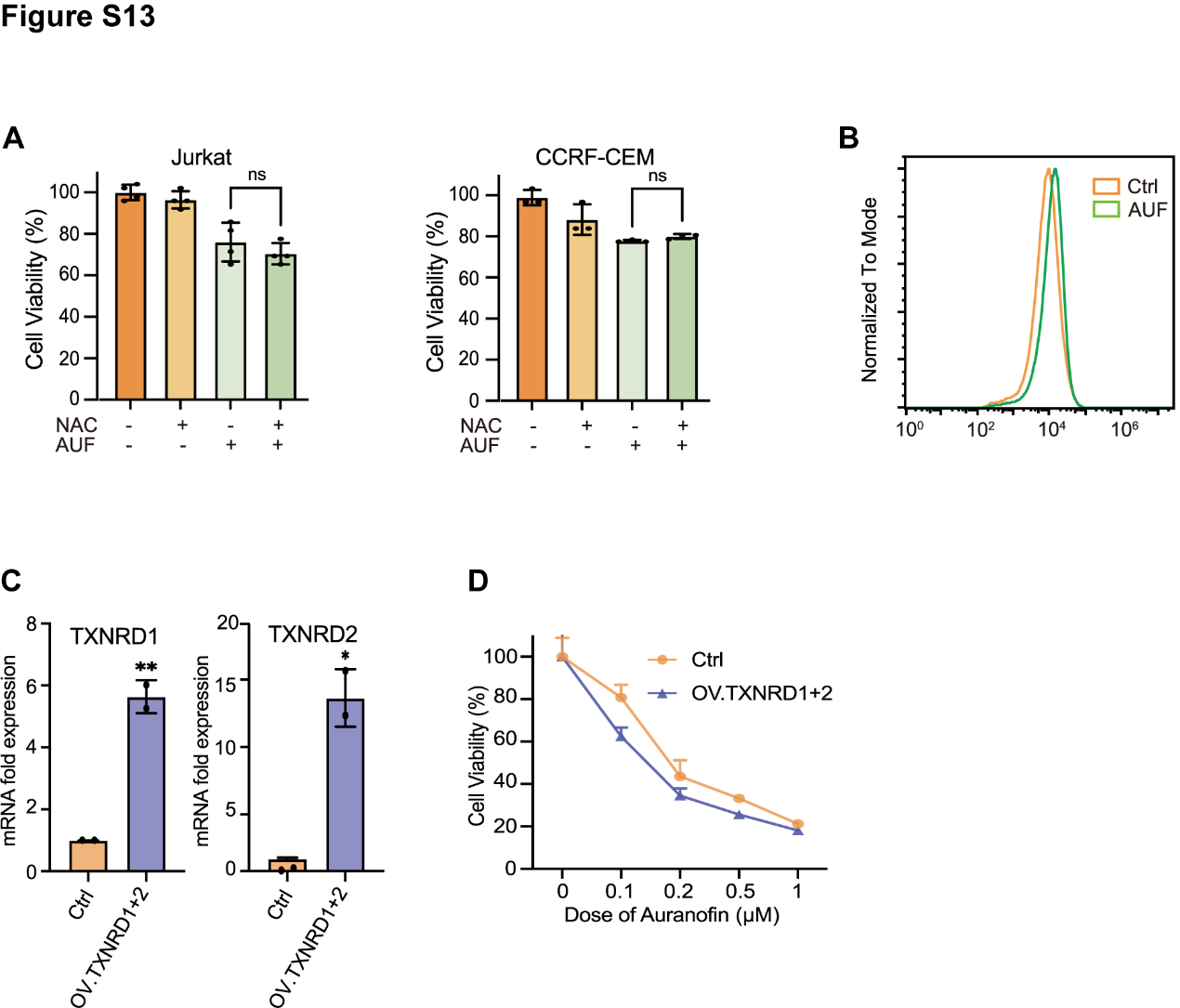


**Figure S13. a.** Jurkat cell viability treated with or without auranofin (0.1 μM) or NAC. NAC did not attenuate auranofin-induced cytotoxicity. Data are the mean ± SD (two-tailed unpaired Student’s t test). **b.** Flow cytometry analysis of ROS level in Jurkat cell lines treated with auranofin (0.1 μM) detected by DCFH-DA staining. **c.** The mRNA levels of TXNRD1 and TXNRD2 in Jurkat cells overexpressing TXNRD1 and TXNRD2. **d.** Cell viability of Jurkat cells treated with various concentrations of auranofin with or without TXNRD1 and TXNRD2 overexpression. Overexpression did not attenuate auranofin-induced cytotoxicity. Data are the mean ± SD. N=3. (Two-tailed unpaired Student’s t test, * *p* < 0.05, ** *p* < 0.01).


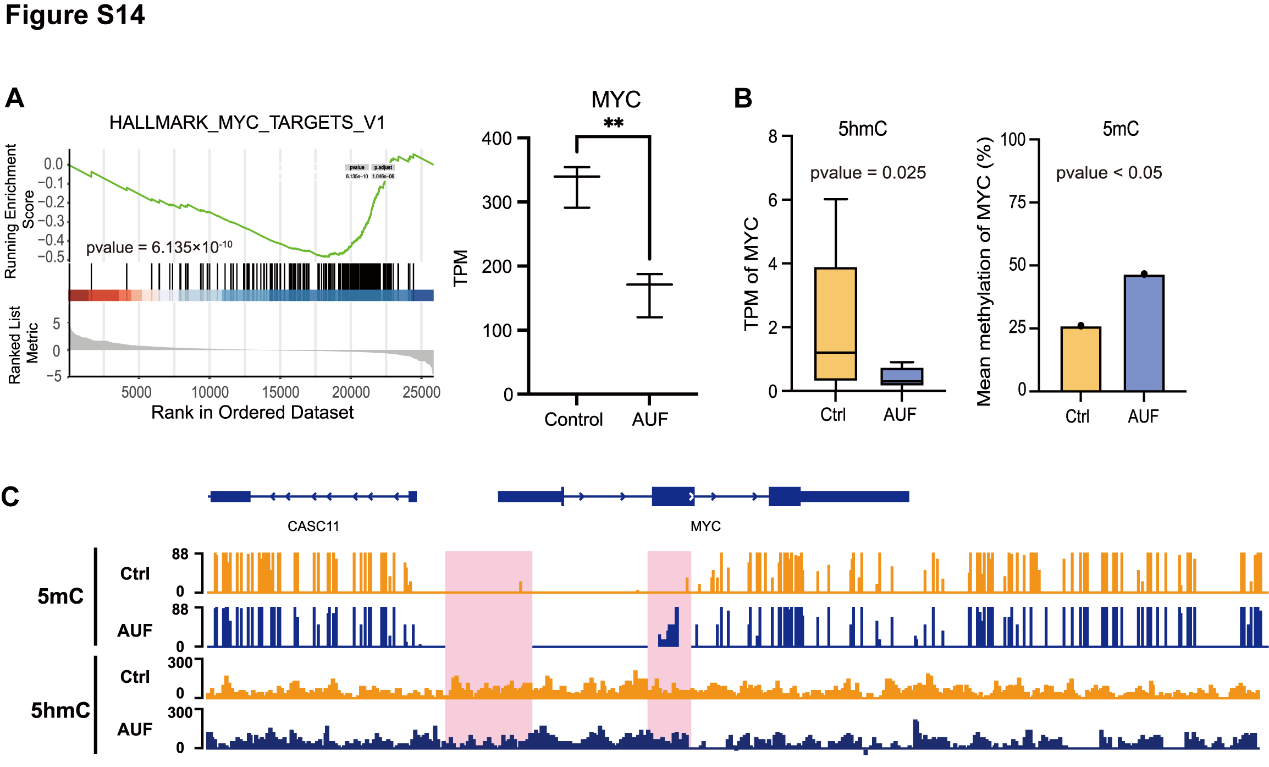


**Figure S14. a.** Gene set enrichment analysis (GSEA) of genes associated with the MYC signaling pathway (left) and transcripts per million (TPM) of c-Myc after auranofin treatment (right) by RNA-seq. Data are the mean ± SD. (Two-tailed unpaired Student’s t test, ** *p* < 0.01). **b.** 5hmC TPM (left) and mean methylation (%) (right) of c-Myc after auranofin treatment from 5hmC-Seal and WGBS. **c.** 5hmC and 5mC signals around the chromosomal region of c-Myc.


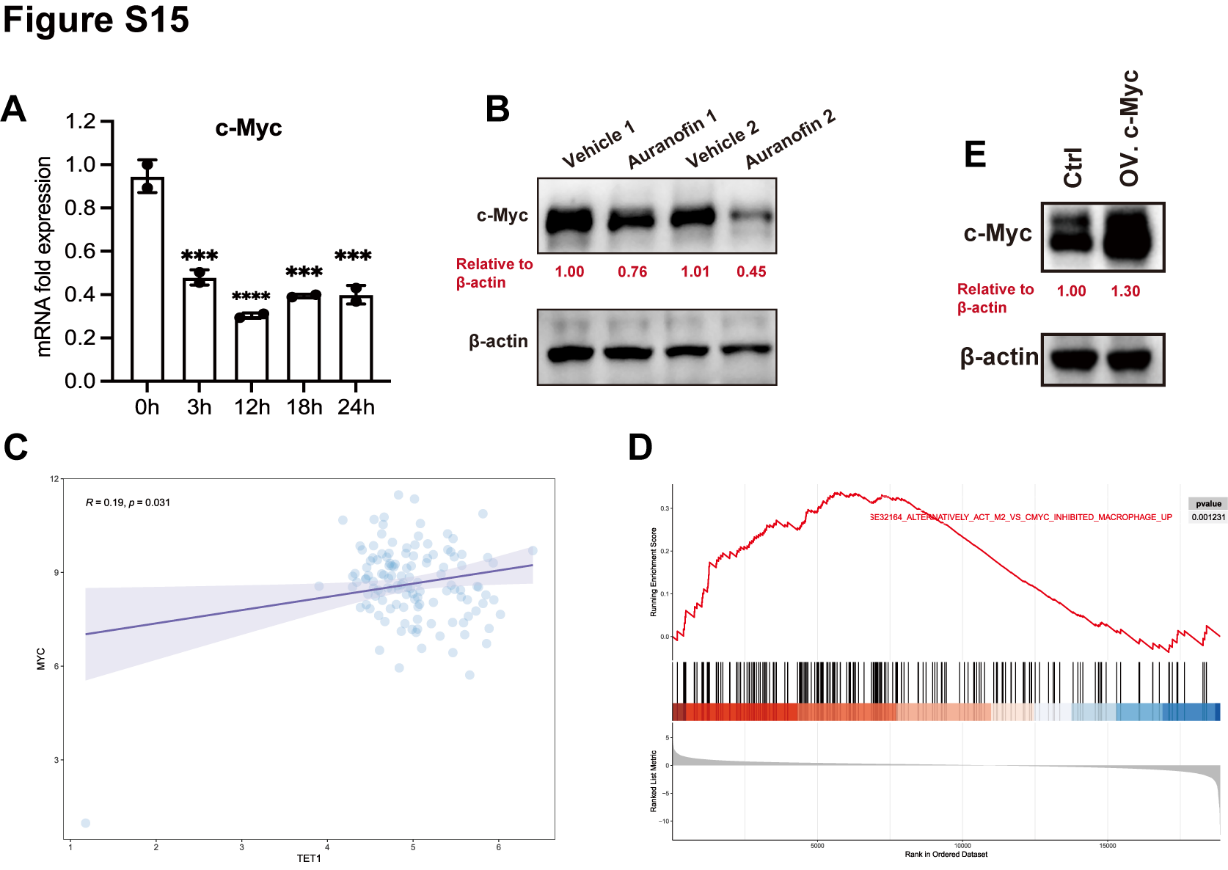


**Figure S15. a.** Time-lapse analysis of the mRNA levels of c-Myc in Jurkat cells after auranofin treatment. Data are the mean ± SD. (Two-tailed unpaired Student’s t test, *** *p* < 0.001, **** *p* < 0.0001). **b.** Western blot analysis showing that c-Myc expression was down-regulated in T-ALL cells isolated from CDX mice model treated with auranofin. **c.** Correlation analysis of TET1 and c-Myc expression in T-ALL patients. **d.** GSEA analysis showing that TET1-high expression group significantly differs in the gene-enriched MYC pathway compared with samples from the TET1-low expression group.
